# From Diabetes to Dementia: Identifying Key Genes in the Progression of Cognitive Impairment

**DOI:** 10.3390/brainsci14101035

**Published:** 2024-10-18

**Authors:** Zhaoming Cao, Yage Du, Guangyi Xu, He Zhu, Yinchao Ma, Ziyuan Wang, Shaoying Wang, Yanhui Lu

**Affiliations:** 1 School of Nursing, Peking University, Beijing 100191, China; yuanfang166@163.com (Y.D.); xuguangyi1998@163.com (G.X.); 2 School of Stomatology, Peking University, Beijing 100191, China; zhuhe882@alu.scu.edu.cn (H.Z.); 13247193850@163.com (S.W.); 3 The Key Laboratory of Medical Immunology of the National Health Commission, School of Basic Medical Sciences, Peking University, Beijing 100191, Chinalegolovers@pku.edu.cn (Z.W.)

**Keywords:** chronic illness, clinical decision-making, dementia, diabetes, genetics, genomics, neurology, nursing assessment, weighted gene co-expression network analysis, LASSO, key genes

## Abstract

Objectives: To provide a basis for further research on the molecular mechanisms underlying type 2 diabetes-associated mild cognitive impairment (DCI) using two bioinformatics methods to screen key genes involved in the progression of mild cognitive impairment (MCI) and type 2 diabetes. Methods: RNA sequencing data of MCI and normal cognition groups, as well as expression profile and sample information data of clinical characteristic data of GSE63060, which contains 160 MCI samples and 104 normal samples, were downloaded from the GEO database. Hub genes were identified using weighted gene co-expression network analysis (WGCNA). Protein–protein interaction (PPI) analysis, combined with least absolute shrinkage and selection operator (LASSO) and receiver operating characteristic (ROC) curve analyses, was used to verify the genes. Moreover, RNA sequencing and clinical characteristic data for GSE166502 of 13 type 2 diabetes samples and 13 normal controls were downloaded from the GEO database, and the correlation between the screened genes and type 2 diabetes was verified by difference and ROC curve analyses. In addition, we collected clinical biopsies to validate the results. Results: Based on WGCNA, 10 modules were integrated, and six were correlated with MCI. Six hub genes associated with MCI (TOMM7, SNRPG, COX7C, UQCRQ, RPL31, and RPS24) were identified using the LASSO algorithm. The ROC curve was screened by integrating the GEO database, and revealed COX7C, SNRPG, TOMM7, and RPS24 as key genes in the progression of type 2 diabetes. Conclusions: COX7C, SNRPG, TOMM7, and RPS24 are involved in MCI and type 2 diabetes progression. Therefore, the molecular mechanisms of these four genes in the development of type 2 diabetes-associated MCI should be studied.

## 1. Introduction

**Figure 1:**
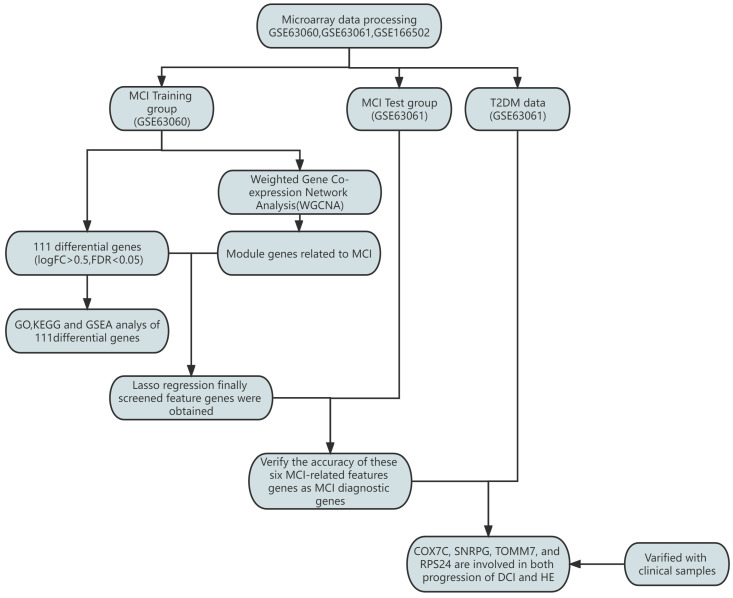
The workflow of the analysis process.

Diabetes rates have been increasing annually in the global population due to lifestyle changes and aging populations in recent years. As reported by the International Diabetes Federation (IDF), approximately 537 million adults (20–79 years old) had diabetes in 2021, with the number predicted to rise to 643 million by 2030 and 783 million by 2045 [1]. Patients with type 2 diabetes account for more than 90% of all diabetes cases [2]. This type of diabetes occurs mainly due to low insulin levels or poor cellular responses to insulin, and it is characterized by hyperglycemia. It is becoming increasingly evident that hyperglycemia can affect the circulatory, nervous, and immune systems [3,4,5]. Cognitive impairment is a common complication of diabetes mellitus and its associated comorbidities. Patients with diabetes mellitus are 1.5–2.0 times more likely to experience cognitive decline, cognitive impairment, or dementia than non-diabetic patients [6]. Mild cognitive impairment (MCI) is an extremely unstable transition state between normal aging and mild Alzheimer’s disease (AD). Type 2 diabetes may be a risk factor for MCI, which increases the risk of AD [7]. The irreversible development of MCI into AD seriously reduces the patient’s quality of life and increases the burden on their families. (Figure 1).

The development of MCI involves the regulation of multiple genes. Prediabetes and diabetes are independently related to an accelerated decline in cognitive ability, especially in older adults [8]. The etiology of type 2 diabetes-associated MCI is multifactorial, and its potential pathological mechanism is not fully understood. Evidence from preclinical and clinical studies supports the viewpoints of insulin resistance (IR), metastatic inflammation, redox deficiency, cerebral microvascular dysfunction, and intestinal strain imbalance [9]. To the best of our knowledge, neuroimaging, cerebrospinal fluid, and blood samples are currently used to facilitate the early identification and clinical intervention of AD and to distinguish patients with MCI who are at a high risk of developing AD [10]. However, as neuroimaging is expensive and cerebrospinal fluid detection is invasive, neither can be used as a routine clinical route for identifying MCI. In contrast, blood samples have the potential to be used for the early screening and diagnosis of MCI because of their accessibility and non-invasive or minimally invasive nature.

With the development of high-throughput sequencing technology, weighted gene co-expression network analysis (WGCNA) analysis has become a new tool for identifying blood biomarkers [11,12,13]. There have been a few reports on MCI and type 2 diabetes using the WGCNA algorithm. Only one previous study used the least absolute shrinkage and selection operator (LASSO) model of the hub gene to predict AD, which did not involve MCI and thus lacked significance for the early diagnosis of AD [14]. This study aimed to explore the key genes involved in the progression of MCI and type 2 diabetes by mining public databases, constructing a weighted gene co-expression network, and identifying the key gene modules and genes that are closely related to MCI and type 2 diabetes. This could lead to the identification of new target molecules to elucidate the occurrence and progression mechanisms of type 2 diabetes-associated MCI, resulting in new ideas for clinical medication guidance and epidemiological screening.

## 2. Results

### 2.1. Screening of DEGs and Biological and Functional Enrichment Analysis

**Figure 2:**
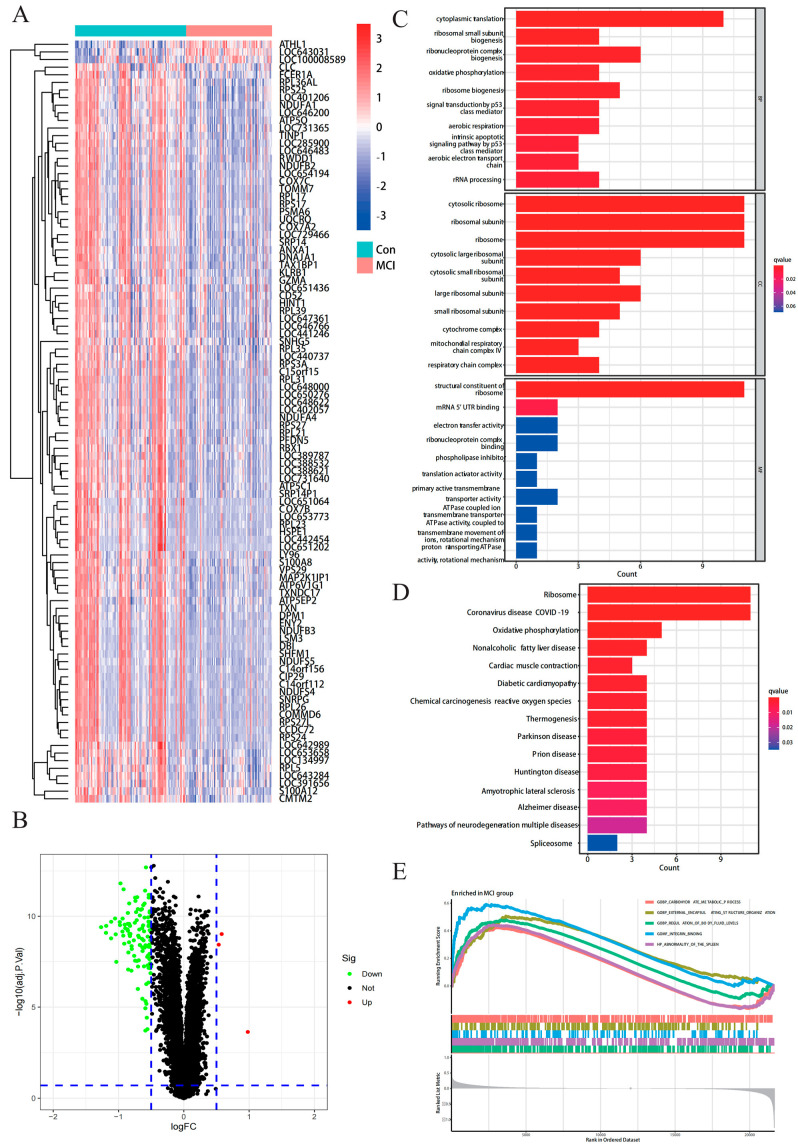
Differentially expressed genes (DEGs) between MCI and normal control samples, and functional enrichment analysis. The heatmap and volcano plot of DEGs were obtained by cluster analysis. (**A**) In the horizontal axis, Con represents the normal control group, MCI represents the cognitive impairment group, and the vertical axis represents the DEGs. (**B**) Volcano plot of differently expressed genes. Red indicates up-regulated genes, and green indicates down-regulated genes. Blue dotted line: Threshold coordinate line (**C**) GO terms. (**D**) KEGG pathways. (**E**) GSEA analysis of DEGs in the MCI group.

The Limma R package identified 111 DEGs between 160 MCI samples and 104 control samples, of which three were upregulated (LOC100008589, ATHL1, and LOC643031) and 108 were downregulated in the MCI samples. A heat map of the DEGs dataset is shown in Figure 2A, and a volcano plot is shown in Figure 2B.

We performed GO functional analysis, KEGG pathway analysis, and gene set enrichment analysis (GSEA) in order to gain further insight into the biological functions of the DEGs. The GO analysis results showed that the DEGs were mainly enriched in the formation of cytoplasmic translation, aerobic electron transport (Biological Process, BP), cytosolic ribosomes (Cellular Component, CC), and structural constituents of ribosomes (Molecular Function, MF) (Figure 2C). The KEGG pathway analysis showed that the DEGs were mainly involved in the ribosome, Coronavirus disease 2019 (COVID-19), oxidative phosphorylation, Parkinson’s disease, non-alcoholic fatty liver disease, prion disease, etc. (Figure 2D). The GSEA analysis demonstrated that DEGs were mainly involved in cytoplasmic translation, ribosomal subunit, ribosomes, and structural constituents of the ribosome in the control group (Figure 2E), and in carbohydrate metabolic processes, external encapsulating structure organization, regulation of body fluid levels, integrin binding, and spleen abnormalities in the MCI group (Figure S1, Supplementary Material).

### 2.2. WGCNA Co-Expression Analysis

**Figure 3:**
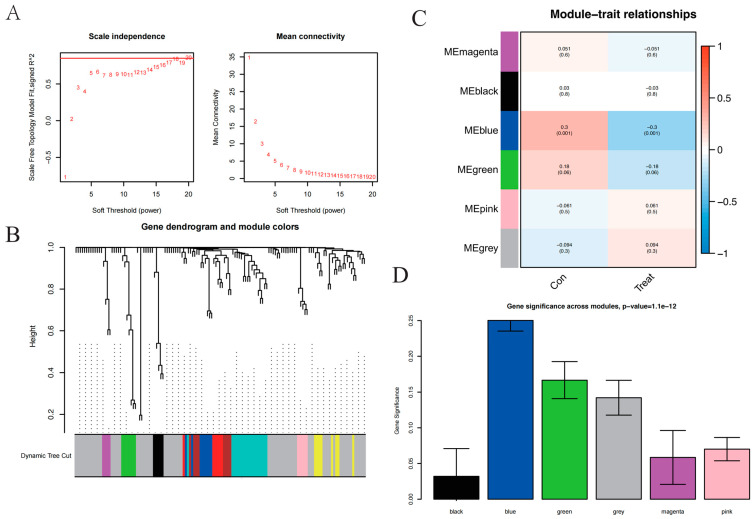

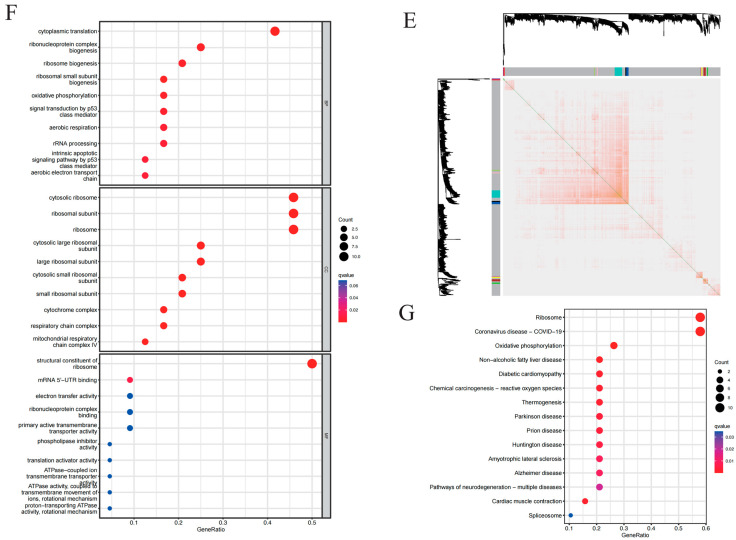
Identification of co-expressed modules and relationship of modules and disease status by WGCNA. (**A**) soft threshold filtering. (**B**) Hierarchical clustering tree of WGCNA co-expression network. (**C**) Correlation between modules and disease status. (**D**) Barplot of mean gene significance (GS) across modules. (**E**) Heatmap plot of coexpressed genes. (**F**) GO terms for biological processes (BP), GO terms for cellular components (CC), GO terms for molecular function (MF). (**G**) KEGG pathways.

Through WGCNA, a co-expression network was constructed, and the co-expression of gene modules was identified using a dynamic tree-cut algorithm. The cluster dendrogram of genes and module–trait relationships are shown in Figure 3. Based on the scale-free network distribution fit, R2 was set to 0.85, and 3 was chosen as the optimal soft threshold (Figure 3A). We calculated the correlation matrices between genes and the TOM and then built a hierarchical clustering tree using the TOM. The branches of the dendrogram corresponded to ten different gene modules (Figure 3B); each leaf on the dendrogram corresponded to a gene, and similar genes were clustered into modules of the same color. Gene modules were identified using the dynamic clipping tree method, and modules with high similarity were merged. Finally, six modules were obtained. A heatmap of the correlation between the modules and disease status is shown in Figure 3C. The results showed that only the blue module was significantly associated with MCI (*p* < 0.01). Similarly, the bar plot of mean gene significance (GS) across modules showed that the blue module was the most significant (Figure 3D). The co-expression network heatmap (Figure 3E) showed that the gene correlation in the blue module was higher than that in the other modules.

### 2.3. Significantly Enriched GO Terms and KEGG Pathways

GO and KEGG pathway enrichment analyses were performed using the cluster profile package in R (16). GO enrichment analysis included Biological Process (BP), Cellular Component (CC), and Molecular Function (MF) terms. GO terms and KEGG pathways with *p* < 0.05 (adjusted) were considered to be significantly enriched. GO analysis and KEGG analysis were performed on the 41 genes in the blue module using the DAVID database. Using the *p* value as the criterion, the results of the GO and KEGG analyses were sorted from low to high, and the top 10 most significant results were selected and plotted using R software. GO analysis (Figure 3F) showed that BP was mainly concentrated in the cytoplasmic translation, ribosomal small subunit biogenesis, ribonucleoprotein complex biogenesis, and oxidative phosphorylation; CC was mainly concentrated in the cytosolic ribosome and ribosomal subunit; MF was mainly concentrated in the structural constituent of the ribosome and mRNA 5’UTR binding. The KEGG pathways (Figure 3G) were mainly involved in the ribosome, COVID-19, oxidative phosphorylation, non-alcoholic fatty liver disease, and diabetic cardiomyopathy.

### 2.4. PPI Network Construction and Key Genes Screening

**Figure 4:**
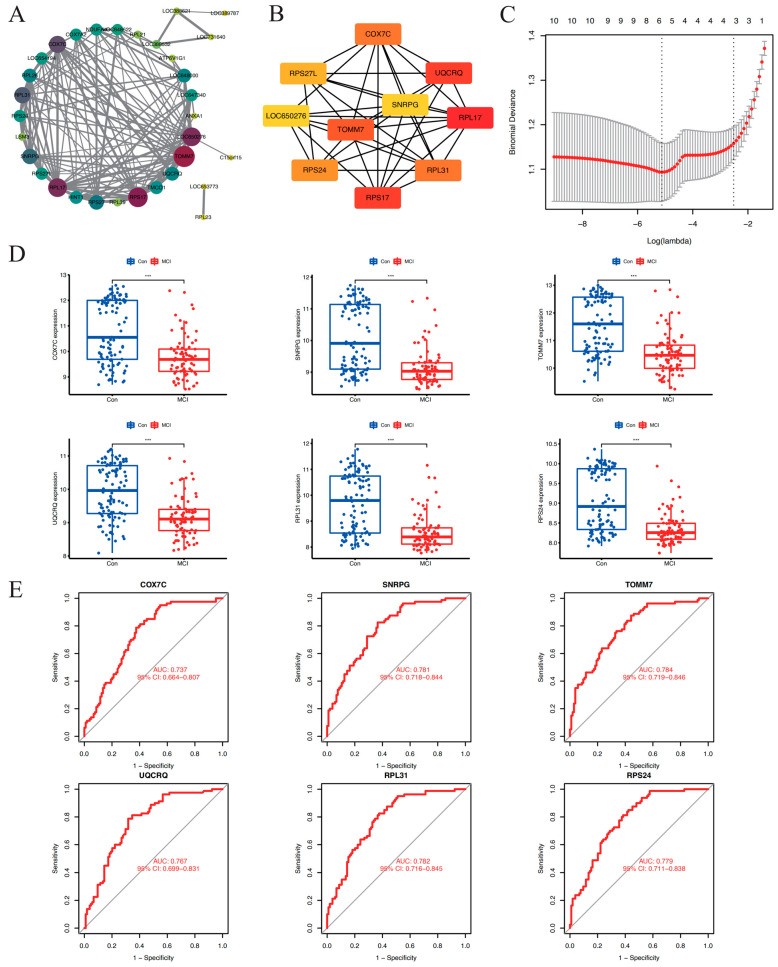
PPI network construction and key genes screening. (**A**), Diagram of protein–protein interaction (PPI) network of the blue module. The degree of the node was reflected by its size and color. The larger the node, the deeper the color from yellow to purple, and the greater the degree. (**B**), the network diagram of the top 10 genes screened via the MCC algorithm. The darker color, from yellow to red, represented a greater score. (**C**), LASSO screening analysis of related genes in the blue module. (**D**), Difference analysis of COX7C, SNRPG, TOMM7, UQCRQ, RPL31, and RPS 24 expressions in MCI and normal control group. (**E**), ROC curve detection of COX7C, SNRPG, TOMM7, UQCRQ, RPL31, and RPS24 to predict the occurrence of MCI. Significance: ***, *p* < 0.001.

The 41 genes in the blue module were selected to construct a PPI network. The interaction pairs with confidence scores ≥ 0.4 were visualized using Cytoscape software (v3.8.2). There was a total of 34 interacting genes (Figure 4A). Cytoscape was used to visualize the PPI network, and the cytoHubba plugin with the MCC algorithm was used to screen the top 10 key genes (Figure 4B). Using LASSO for further analysis, six MCI target genes were screened: homo sapiens translocase of outer mitochondrial membrane 7 (TOMM7), small nuclear ribonucleoprotein polypeptide G (SNRPG), cytochrome oxidase subunit 7C (COX7C), ubiquinol-cytochrome c reductase complex III subunit VII (UQCRQ), ribosomal protein L31 (RPL31), and ribosomal protein S24 (RPS24) (Figure 4C).

### 2.5. Validation of the Screened Genes

Compared to the control group, the expression of COX7C, SNRPG, TOMM7, UQCRQ, RPL31, and RPS24 was downregulated in the MCI group (Figure 4D). ROC curve analysis was used to further assess the ability of the six genes to determine the occurrence of MCI (Figure 4E). The areas under the curve (AUC) for predicting the occurrence of MCI were COX7C (AUC = 0.737; 95% confidence interval (95% CI): 0.663–0.803); SNRPG (AUC = 0.781; 95% CI: 0.718–0.844); TOMM7 (AUC = 0.784; 95% CI: 0.719–0.846); UQCRQ (AUC = 0.767; 95% CI: 0.699–0.831), RPL31 (AUC = 0.782; 95% CI: 0.716–0.845), RPS24 (AUC = 0.779; 95% CI: 0.711–0.838). These results indicate that COX7C, SNRPG, TOMM7, UQCRQ, RPL31, and RPS24 could predict the occurrence of MCI.

### 2.6. The Diagnostic Value of MCI Key Genes in Type 2 Diabetes

**Figure 5:**
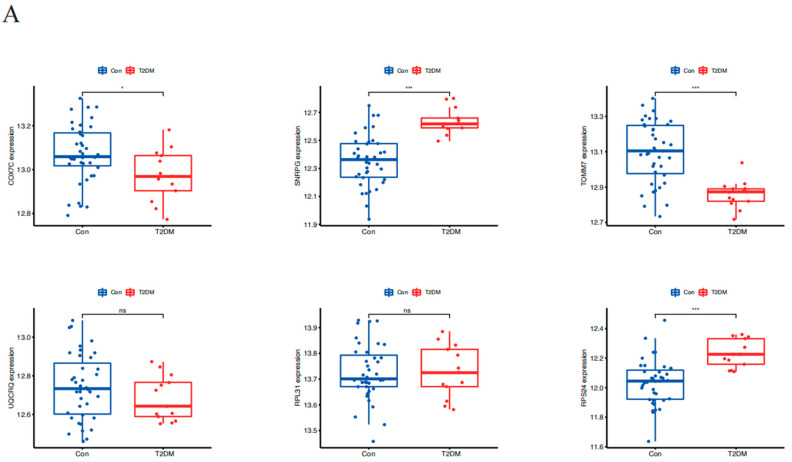

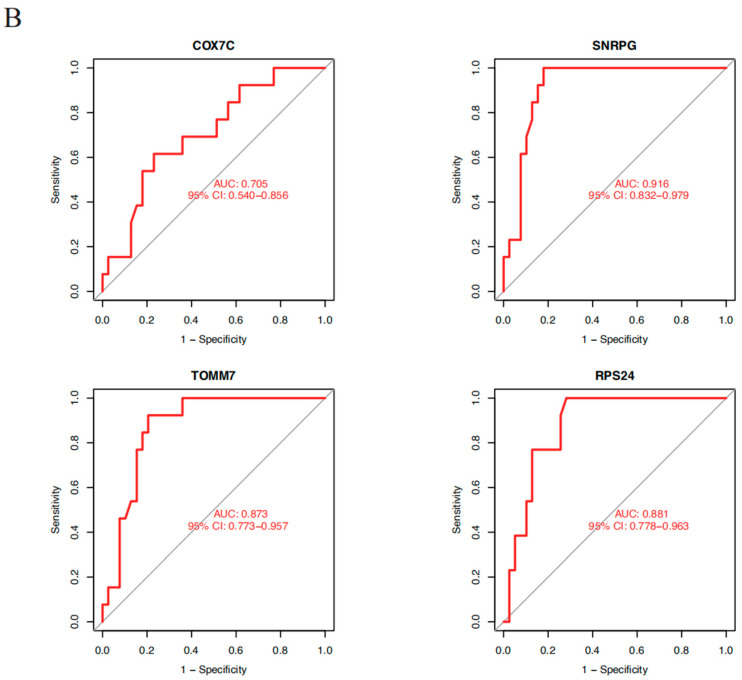
(**A**), Difference analysis of COX7C, SNRPG, TOMM7, UQCRQ, RPL31, and RPS24 expression in type 2 diabetes and normal groups. (**B**), ROC curve detection of COX7C, SNRPG, TOMM7, and RPS24 to predict the occurrence of type 2 diabetes. Significance identification: ns, *p* ≥ 0.05; *, *p*< 0.05; ***, *p* < 0.001.

The t-test results showed that the expression levels of four genes (COX7C, SNRPG, TOMM7, and RPS24) were significantly different between the type 2 diabetes and control groups (*p* < 0.05) (Figure 5A). The ROC curve was used to verify the diagnostic value of these four genes in type 2 diabetes (Figure 5B). The results were COX7C (AUC = 0.705; 95% CI: 0.540–0.854), SNRPG (AUC = 0.916; 95% CI: 0.832–0.980), TOMM7 (AUC = 0.873; 95% CI: 0.769–0.959), and RPS24 (AUC = 0.881; 95% CI: 0.778–0.963).

### 2.7. Validation of Gene Expression Levels

**Figure 6:**
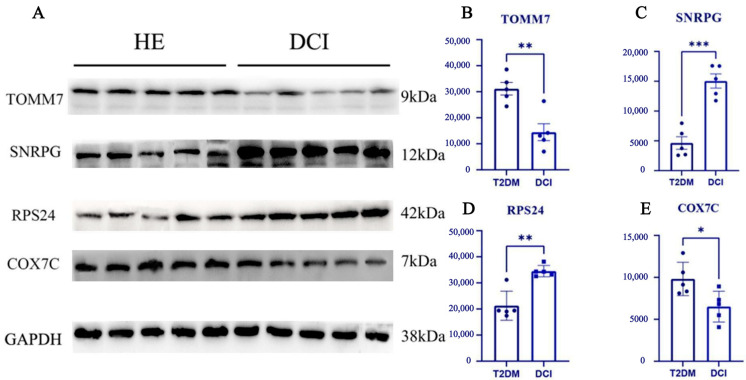
(**A**) Validation of 4 diagnostic feature biomarkers in clinical tissues via Western blot analysis. The expression of (**B**), TOMM7, (**C**), SNRPG, (**D**), RPS24, and (**E**), COX7C. Statistical significance: *p < 0.05; **p < 0.01; ***p < 0.001.

The Western blot results indicated that the expression levels of TOMM7, SNRPG, RPS24, and COX7C in the peripheral blood serum of both groups aligned with the findings of our database analysis. Furthermore, there was a notable disparity in the expression of these proteins between the two groups based on peripheral blood samples (Figure 6).

## 3. Discussion

We used WGCNA combined with the LASSO algorithm to screen the characteristic genes for MCI and then evaluated their ability to predict the occurrence of type 2 diabetes [15]. The advantage of this method is that it ensures that the results guide clinical genetic screening for the occurrence of MCI in patients with type 2 diabetes sensitivity, which is also known as the true positive rate [16]. Functional enrichment analysis revealed that DEGs were mostly enriched in the cytosolic ribosome, SRP-dependent cotranslational proteins targeting the membrane, oxidative phosphorylation, ribosomal structure, Parkinson’s disease, AD, and metabolism. This is consistent with the results of previous studies [17,18,19,20]. The main functions of the gene set enriched in the control group were cytoplasmic translation, cytosolic ribosome, ribosomal subunit, ribosome, and structural constituents of the ribosome, whereas the main gene sets and functions in the MCI group were carbohydrate metabolic process, external encapsulating structure organization, regulation of body fluid levels, and integrin binding. These results also confirm that MCI progression is likely associated with abnormal ribosome function and energy metabolism. 

Previous studies have confirmed overlapping features of MCI and type 2 diabetes in cell metabolism, biological regulation, and other gene functions [21,22,23]. From a metabolic perspective, MCI is considered a fluctuation between high- and low-energy states [24]. Therefore, MCI development is closely related to energy metabolism in the brain. Studies have confirmed that the systemic insulin resistance observed in type 2 diabetes is reflected in the inability of cells to respond appropriately to insulin signals in the brain, which is manifested by impairment in cognitive areas such as executive function, memory, and processing speed [25]. This study was based on the GEO database from GSE63060, which contains whole-blood mRNA expression profile data; 160 MCI patients and 104 healthy participants matched for age and sex were included. First, six modules related to the MCI phenotype were obtained by WGCNA analysis, among which the blue module was the most related to MCI. Genes in the blue module were further analyzed using GO and KEGG. GO analysis showed that BP was mainly concentrated in cytoplasmic translation, ribosomal small subunit biogenesis, ribonucleoprotein complex biogenesis, and oxidative phosphorylation; CC was mainly concentrated in the cytosolic ribosome and ribosomal subunit. Ribosomes are essential for protein synthesis, and their dysfunction can lead to widespread cellular abnormalities. In the context of T2D-related MCI, abnormal ribosome function could contribute to cognitive impairment through several mechanisms, such as impaired protein synthesis, endoplasmic reticulum (ER) stress, altered insulin signaling, and mitochondrial dysfunction. These findings highlight the complex interplay between ribosomal function, metabolic regulation, and cognitive health, suggesting that targeting ribosomal pathways could be a novel approach to preventing or treating T2D-related MCI; MF was mainly concentrated in the structural constituent of the ribosome and mRNA 5’UTR binding. The KEGG pathways involved were mainly ribosomes, COVID-19, oxidative phosphorylation, non-alcoholic fatty liver disease, and diabetic cardiomyopathy.

It is worth noting that the GO and KEGG enrichment results suggest that the process of MCI is closely related to the operation of ribosome generation. The ribosome is a complex collection of ribosomal RNA (rRNA) and ribosomal proteins responsible for RNA translation into proteins in an organism. The Molecular Function section of the GO enrichment results suggests that MCI signature genes are concentrated at the 5’UTR end of mRNA, which is critical in the mRNA translation process, and its roles include ribosome recruitment, scanning and initiation codon selection, and maintenance of mRNA stability [25,26]. In recent years, more and more studies have confirmed that the 5’UTR end also plays an essential role in post-transcriptional modifications. Zhang et al. [24] used bioinformatics analysis to reveal a unique role of 5’UTR-specific m^6^A RNA modifications in mouse cerebral cortex development, and numerous studies have confirmed that the results of post-transcriptional modifications in the normal development of neuronal cells and cellular energy metabolism processes play an important role.

Using the WGCNA and LASSO algorithms, we identified six signature genes in the development of MCI. In addition, we verified that four of these genes (COX7C, SNRPG, TOMM7, and RPS24) are also able to predict the risk of type 2 diabetes and can be used as key genes in type 2 diabetes and MCI. Although the mechanisms underlying MCI in patients with type 2 diabetes are currently unclear, accumulating evidence suggests that insulin resistance (IR), neuroinflammation, oxidative stress, cerebral microvascular dysfunction, lymphatic dysfunction, metal ion disorders, and gut dysbacteriosis are involved in type 2 diabetes-associated MCI [26]. Direct and indirect evidence supported the expression of most of the four genes.

First, COX7C is a protein-coding gene that has only been shown to be related to pathways, including complex mitochondrial assembly and ATP synthesis, plays an essential role in cellular energy metabolism, and is a potential biomarker for diabetes-related sepsis (DRS) [27]. In contrast to other mRNAs that do not encode the mitochondrial protein [28], COX7C was preferentially associated with mitochondria in a mouse neural cell line and in mouse primary motor neuron axons [29]. The altered expression of COX7C in our study suggests that impaired energy metabolism may be a key factor in T2D-related MCI, potentially offering a target for therapeutic interventions aimed at improving mitochondrial function.

Second, the protein encoded by the SNRPG gene is a component of the U1, U2, U4, and U5 small nuclear ribonucleoprotein complexes, which are precursors of the spliceosome. The encoded protein may also be part of the U7 small nuclear ribonucleoprotein complex, which participates in the processing of the 3’ end of histone transcripts and plays a vital role in the progression of AD [21,22]. Previous studies have highlighted the role of this gene in the development of neuropsychological diseases. Downregulation of SNRPG by risperidone affects protein synthesis, which in turn affects the mitogenic signal [30]. Neuropsychological illnesses, such as schizophrenia, which regulate reward-related behavior by activating mitogen-activated protein kinase (MAPK), have been linked to functional impairments of dopamine. Funahashi et al. [25] performed a proteomic analysis in mice to identify, through gene expression, the transcription factors relevant to MAPK-regulated reward-related learning and memory and screened more than 400 cyclic adenosine monophosphate response element binding protein-binding protein (CBP)-interacting proteins, including SNRPG; however, the exact mechanisms require further research. Another study identified the co-expressive m6A regulators SNRPG and SNRPD2 as potential biomarkers for predicting the progression from MCI to AD [30]. Its role in T2D-related MCI may be linked to aberrant protein synthesis and processing, which are critical for maintaining neuronal health and function. The identification of SNRPG as a key gene in our study highlights the potential importance of RNA processing in the pathogenesis of T2D-related cognitive decline.

Third, the TOMM7 gene encodes a subunit of the mitochondrial outer membrane translocase, a protein that regulates the assembly and stability of the translocase complex. A previous study confirmed a link between the TOMM7 gene and type 2 diabetes, in which genetic variation at the rs2240727 locus was associated with type 2 diabetes in the Chinese Dong population [31]. Several studies have supported the association between TOMM7 and degenerative diseases. One study identified that the outer mitochondrial membrane translocase (TOM complex) is a key determinant of mitochondrial homeostasis, and mitochondrial dysfunction may contribute to the development of sporadic neurodegenerative diseases [32]. TOMM7 plays a crucial role in stabilizing PINK1 on the outer mitochondrial membrane and responding to mitochondrial damage [33], which may influence mitochondrial autophagy, whereas PINK1 may act in a quality control pathway, preventing the accumulation of dysfunctional mitochondria in Parkinson’s disease [34]. Another study demonstrated that TOMM7 is a key regulator of cerebrovascular homeostasis and that TOMM7 deletion promotes the mitochondrial import of Rac1 and Rac1-coupled signaling pathways in endothelial cells, impairing cerebral vascular neogenesis and cerebrovascular network formation. Furthermore, targeting the mitochondrial Tomm7-Rac1 axis may be an alternative therapeutic approach for treating neurovascular diseases [35]. Moreover, Hasson et al. demonstrated that TOMM7 is essential for stabilizing PINK1 on the outer mitochondrial membrane following mitochondrial damage and dysfunction, which contributes to the molecular pathogenesis of neurodegenerative diseases [33]. Taken together, our results, which showed that TOMM7 was downregulated in the MCI group, suggesting that this gene is a key gene in energy metabolism and is involved in the formation of the cerebrovascular network, are consistent with those of previous studies. Cerebral microvascular dysfunction caused by TOMM7 deficiency is the pathophysiological mechanism underlying the progression of type 2 diabetes-associated MCI and AD. This finding underscores the potential of targeting mitochondrial function and vascular health in managing T2D-related MCI.

Fourth, the RPS24 gene encodes a ribosomal protein component of the 40S subunit. This protein belongs to the S24E family of ribosomal proteins. RPS24 is involved in eukaryotic selenoprotein synthesis and regulates free radical metabolism, antioxidant function, immune function, reproductive function, apoptosis, and hormone secretion [36]. However, to the best of our knowledge, no previous studies have reported the involvement of RPS24 in the pathogenesis of MCI or AD. Considering the decreased expression of RPS24 in MCI and the reduction of ribosomal function and protein synthesis, which impair cognitive function, we hypothesized that RPS24 might promote the expression of a ribosomal protein in neurons, affecting protein synthesis. Furthermore, RPS24 is expected to become a new biomarker for screening patients with type 2 diabetes who are more likely to progress to MCI and guide clinical treatment.

However, the peripheral blood expression levels of these genes need to be further verified. Due to the lack of peripheral blood sequencing data for type 2 diabetes and control groups, this study could only select the sequencing data of muscle samples as early biomarkers. Therefore, the efficacy of diagnosing type 2 diabetes-associated MCI needs to be confirmed in large-scale clinical studies.

## 4. Materials and Methods

### 4.1. Data Processing

The Strawberry Perl software (version 5.32. 1. 1), R software (version 4.0.2), and Bioconductor packages (“http://www.bioconductor.org/” (accessed on 5 April 2023)) were used for data processing. Data pre-processing was performed as follows: the homogenized expression profile was downloaded, and the probe identification numbers were converted into official gene symbols according to the GPL6947 and GPL10558 platforms. No-load probes were removed when multiple searches corresponded to the same gene. The median number was selected as the expression level of the gene, and the final expression matrix was used for subsequent analyses. Finally, target gene expression profile data were obtained based on the gene expression spectrum data and protein-coding genes from the Ensembl database.

### 4.2. Differential Expression Analysis

Differentially expressed genes (DEG) between the MCI and control groups were analyzed using the Limma software (V:3.60.4) package in R. DEGs were screened on the standards of |log2FC| > 0.5 and *p* < 0.05 (PFDR < 0.05) after multiple testing adjusted by the method of Benjamini and Hochberg (BH) [14]. A total of 111 DEGs were identified.

### 4.3. Analysis of WGCNA Co-Expression

The WGCNA package in R software was used to construct a gene co-expression network to identify meaningful gene modules. In total, 264 samples from the MCI and control groups were subjected to WGCNA. A correlation matrix was established in the model, and the adjacency relationships of all genes were calculated to determine the size of the soft threshold. To measure the network connectivity between genes, the disordered adjacency relationship between genes was truncated and converted into a topological overlap matrix (TOM) according to the size of the soft threshold. Hierarchical clustering was performed according to the degree of TOM difference, and genes with a similar degree of difference in gene expression were incorporated into the module-eigengenes (ME) of the same module. The correlation between each module and the sample was then calculated using the Pearson correlation analysis coefficient, and the module with the largest correlation coefficient was obtained by accumulating the absolute values. Eventually, highly co-expressed gene modules were inferred from the DEGs, which were divided into modules of different colors. The R package was used to visualize the module results. Gene expression values were extracted from the most significant correlation module for further analysis. 

### 4.4. Gene Ontology (GO) Functional and Kyoto Encyclopaedia of Genes and Genomes (KEGG) Pathway Analysis

To understand the functionalities and pathways involved in the module genes, all genes in the module were screened for related functions and pathways using GO and KEGG analyses.

### 4.5. Protein–Protein Interaction (PPI) Analysis Combined with the LASSO Algorithm for Core Gene Screening and Validation

To understand the potential functions of core genes in patients with MCI, the STRING website (https://string-db.org/ (accessed on 4 May 2023)) was used to conduct a PPI analysis of all genes in this module, and further analysis was performed using the maximal clique centrality (MCC) analysis method in the CytoHubba plug-in in Cytoscape software. At the same time, the LASSO regression analysis was used to analyze the core genes in the screening module. R software was used for further differential analysis, and a receiver operating characteristic (ROC) curve was constructed for verification.

### 4.6. Analysis of the Role of MCI Key Eigengenes in Type 2 Diabetes

The Student’s *t*-test was used to analyze the differences in MCI eigengenes between the type 2 diabetes and control group samples; *p* < 0.05 was considered statistically significant. A ROC curve was constructed to evaluate the ability of the differentially expressed genes to predict the occurrence of type 2 diabetes.

### 4.7. Data and Resource Availability

All MCI mRNA expression data were downloaded from the Gene Expression Omnibus database (GEO, https://www.ncbi.nlm.nih.gov/geo/ (accessed on 4 May 2023)). The GSE63060 (ftp://ftp.ncbi.nlm.nih.gov/geo/series/GSE63nnn/GSE63060/ (accessed on 4 May 2023)) dataset included 160 blood samples from patients with MCI and 104 from healthy controls. The mRNA expression data of this dataset were analyzed using the Illumina HumanHT-12 V3.0 expression BeadChip platform. GSE166502 (ftp.ncbi.nlm.nih.gov/geo/series/GSE166nnn/GSE166502/matrix/ (accessed on 4 May 2023)) includes 13 muscle cell samples from 13 patients with type 2 diabetes and 13 healthy controls. The mRNA expression data for this dataset were obtained using the Illumina HumanHT-12 BeadChip platform. 

### 4.8. Ethical Considerations

This study was conducted after ethical approval was obtained. The study protocol was approved by the Ethics Committee of Peking University (No. 2022016); the approval date (3 April 2022) and the Declaration of Helsinki were strictly adhered to during the conduct of this study. All participants in this study signed an informed consent form.

### 4.9. Clinical Specimens

In May 2023, ten blood samples were collected in Tianjin, China. Five of these patients with clinically confirmed type 2 diabetes mellitus (T2DM) combined with new-onset cognitive impairment were included in the diabetic cognitive impairment (DCI) group. Sex- and age-matched healthy persons served as controls (Con).

### 4.10. Plasma Protein Extraction and Western Blot

Plasma proteins were extracted using the Solarbio Plasma Protein Extraction Kit (Cat#EX1170, Solarbio, Beijing, China) as follows: For every 200 μL of protein extraction solution, 2 μL of protease inhibitor mixture was added, followed by mixing and incubation on ice. The whole blood sample was centrifuged at 4 °C, 1500× *g*, for 10 min, and the cell precipitate was discarded. The supernatant plasma was mixed with 100–200 μL of protein extract for every 100 μL of plasma sample and incubated at 4 °C for 5 min, followed by centrifugation at 4 °C, 14,000× *g*, for 10 min. The supernatant was pipetted into another clean pre-cooled centrifuge tube to obtain plasma proteins. The protein concentration was determined using the BCA Protein Assay Kit (Cat#PC0020, Solarbio) according to the manufacturer’s instructions. Equal quantities of total protein were electrophoresed and transferred to PVDF membranes. After blocking and incubation with primary antibodies, the membranes were incubated with corresponding secondary antibodies. The membranes were then washed and visualized using an enhanced chemiluminescence kit, and the integrated optical density of the protein bands was analyzed using ImageJ software (v.1.53).

## 5. Conclusions

We found potential genetic biomarkers for MCI in patients with type 2 diabetes using WGCNA combined with the LASSO algorithm and further screened four genes, COX7C, SNRPG, TOMM7, and RPS24, which are associated with the occurrence of type 2 diabetes. These genes were significantly enriched in cytoplasmic translation and oxidative phosphorylation and concentrated at the 5’UTR end of mRNA, which is critical in the mRNA translation process. These findings provide additional information on the complex mechanisms underlying the interactions between type 2 diabetes and neurodegenerative disorders.

For type 2 diabetes-associated MCI to be diagnosed early, simple, rapid, and inexpensive detection methods must be identified. Using the key genes in this study for early screening in these high-risk groups can potentially lead to effective, targeted interventions to improve the quality of life of patients with type 2 diabetes and MCI and reduce the burden on their families and caregivers.

## Data Availability

The data supporting the findings of this study are openly available in the GENE EXPRESSION OMNIBUS(GEO) at https://www.ncbi.nlm.nih.gov/geo/ (accessed on 4 May 2023).

## Supplementary Materials

The following supporting information can be downloaded at: https://www.mdpi.com/article/10.3390/brainsci14101035/s1, Figure S1: GSEA analysis of DEGs in the control group.
