# Supplementary material for: From Diabetes to Dementia: Identifying Key Genes in the Progression of Cognitive Impairment
Source: Brain Sci. 2024 Oct 18;14(10):1035. doi: 10.3390/brainsci14101035 (PMC11506463; doi:10.3390/brainsci14101035)
Supplement: Supplementary file 1 [file brainsci-14-01035-s001.zip › brainsci-3240140-supplementary.pdf]

Supplementary Material

From Diabetes to Dementia: Identifying Key Genes in the Progression of Cognitive Impairment

Supplementary Figure

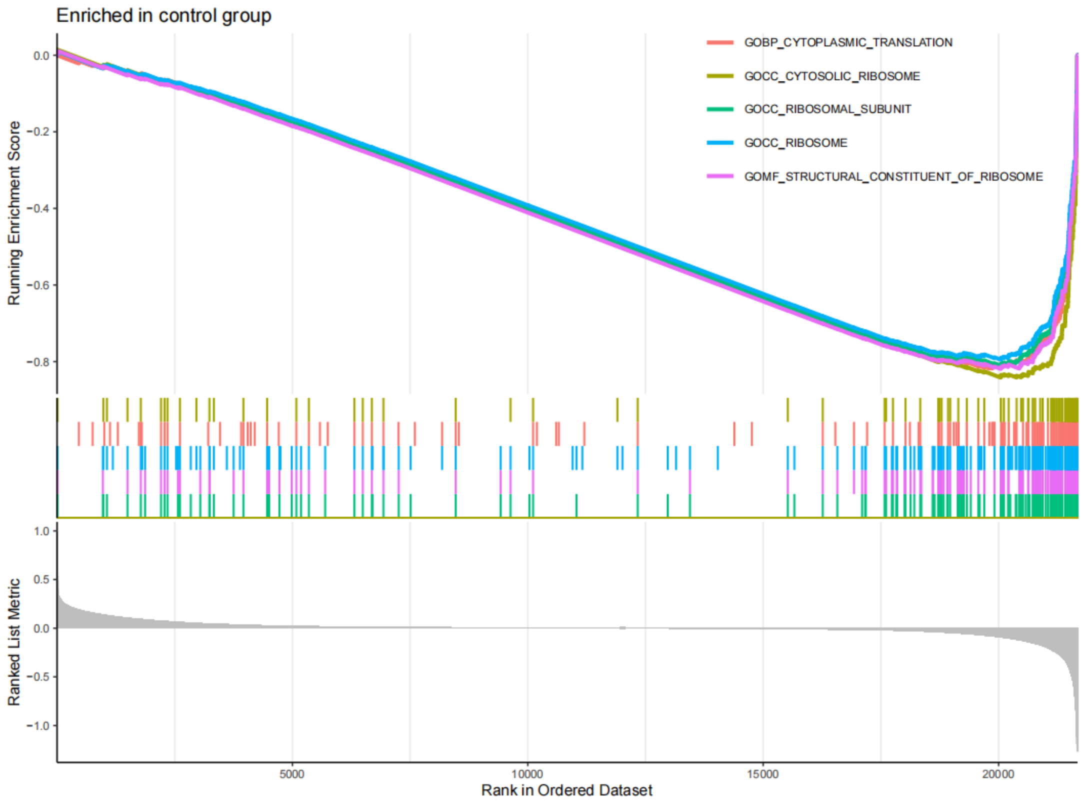

Supplementary Figure S1. GSEA analysis of DEGs in the control group.
